# The spike glycoprotein of highly pathogenic human coronaviruses: structural insights for understanding infection, evolution and inhibition

**DOI:** 10.1002/2211-5463.13454

**Published:** 2022-06-25

**Authors:** Shuyuan Qiao, Shuyuan Zhang, Jiwan Ge, Xinquan Wang

**Affiliations:** ^1^ The Ministry of Education Key Laboratory of Protein Science, Beijing Advanced Innovation Center for Structural Biology, Beijing Frontier Research Center for Biological Structure, School of Life Sciences Tsinghua University Beijing China

**Keywords:** evolution, highly pathogenic coronaviruses, infection, inhibition, spike glycoprotein, structural insights

## Abstract

Highly pathogenic human coronaviruses (CoV) including SARS‐CoV, MERS‐CoV and SARS‐CoV‐2 have emerged over the past two decades, resulting in infectious disease outbreaks that have greatly affected public health. The CoV surface spike (S) glycoprotein mediates receptor binding and membrane fusion for cell entry, playing critical roles in CoV infection and evolution. The S glycoprotein is also the major target molecule for prophylactic and therapeutic interventions, including neutralizing antibodies and vaccines. In this review, we summarize key studies that have revealed the structural basis of S‐mediated cell entry of SARS‐CoV, MERS‐CoV and SARS‐CoV‐2. Additionally, we discuss the evolution of the S glycoprotein to realize cross‐species transmission from the viewpoint of structural biology. Lastly, we describe the recent progress in developing antibodies, nanobodies and peptide inhibitors that target the SARS‐CoV‐2 S glycoprotein for therapeutic purposes.

## Graphical abstract

This review mainly focuses on structural insights into the spike glycoproteins of highly pathogenic coronaviruses, especially SARS‐CoV‐2. This includes the characteristics of the spike glycoprotein, the mechanisms of SARS‐CoV‐2 entry into host cells, the evolution strategies SARS‐CoV‐2 uses to enlarge host range and escape from host immunity, and anti‐spike therapies to fight against SARS‐CoV‐2.

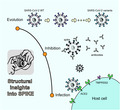

**Abbreviations**
6HB: six‐helix bundle
BH: β‐hairpin
CH: central helix
COVID‐19: coronavirus disease 2019
CoVs: coronaviruses
CR: connecting region
cryo‐ET: cryoelectron tomography
CT: cytoplasmic tail
CTD: C‐terminal domain
E: envelope
FP: fusion peptide
GD: Guangdong
GX: Guangxi
hACE2: human ACE2
HCoV: human coronavirus
HR1: heptad repeat 1
HR2: heptad repeat 2
LA: linoleic acid
MERS‐CoV: Middle East respiratory syndrome coronavirus
N: nucleocapsid
NTD: N‐terminal domain
PCoV: pangolin CoV
RBD: receptor‐binding domain
RBM: receptor‐binding motif
RMSD: root mean square deviation
S: spike
SARS‐CoV‐2: severe acute respiratory syndrome coronavirus 2
SD: subdomain
SPA: single‐particle analysis
TM: transmembrane domain
TMPRSS2: transmembrane serine protease 2
UH: upstream helix
VOCs: variants of concern

Since the initial outbreak at the end of 2019, the coronavirus disease 2019 (COVID‐19) caused by severe acute respiratory syndrome coronavirus 2 (SARS‐CoV‐2) has posed a severe threat to public health and the global economy [1, 2, 3]. As of June 2022, there have been more than 500 million confirmed infections and more than 6 million deaths worldwide (https://covid19.who.int/). In addition to SARS‐CoV‐2, there are at least six other currently known coronaviruses (CoVs) that are able to infect humans: Human coronavirus (HCoV)‐NL63, HCoV‐229E, HKU1, HCoV‐OC43, SARS‐CoV and Middle East respiratory syndrome coronavirus (MERS‐CoV). HCoV‐OC43 and HCoV‐229E were first identified in the 1960s [4, 5] while HCoV‐NL63 and HKU1 were reported in 2004 and 2005, respectively [6, 7]. Infections with these viruses only cause mild and self‐limiting respiratory tract symptoms. Therefore, the unexpected emergence of SARS‐CoV, responsible for the SARS pandemic in 2002–2003 with a fatality of ~ 10% [8], completely changed our views on coronaviruses. Just 10 years later in 2012, a limited outbreak of a second highly pathogenic coronavirus, MERS‐CoV, occurred, with a high fatality rate of ~ 35% [9]. Close to 20 years after SARS, the third highly pathogenic coronavirus, SARS‐CoV‐2, was reported, and the COVID‐19 pandemic is still raging around the world. These three highly pathogenic coronaviruses highlight the threats we face from newly emerging viruses in the 21st century.

CoVs are enveloped, positive‐sense, single‐stranded RNA viruses that can infect humans and animals. They have the largest genomes among RNA viruses, ranging from 27 to 32 kb. There are four CoV genera: *Alphacoronavirus*, *Betacoronavirus*, *Gammacoronavirus* and *Deltacoronavirus*. HCoV‐229E and HCoV‐NL63 are both members of the *Alphacoronavirus* genus. The *Betacoronavirus* genus is further divided into the four subgenera *Embecovirus, Sarbecovirus, Merbecovirus* and *Nobecovirus*. HCoV‐OC43 and HKU1 are members of *Embecovirus*; SARS‐CoV and SARS‐CoV‐2 are members of *Sarbecovirus*; and MERS‐CoV belongs to *Merbecovirus*. CoV genomes encode structural, non‐structural and accessory proteins. The four structural proteins are spike (S), envelope (E), membrane (M) and nucleocapsid (N). The N protein is found within the viral particle, in complex with the viral RNA genome, thus promoting genome stability. As components of the viral envelope, the E and M proteins are both involved in virus assembly. The S glycoprotein protrudes from the surface of the viral envelope and can interact with receptors on host cells, mediating the virus‐cell membrane fusion by which viral entry occurs. Therefore, the S glycoprotein is a critical player in the virulence, tissue tropism and host range of CoVs. The S glycoprotein is also the major target of the host immune response and, consequently, of immense interest in the development of vaccines and therapeutic interventions. In this review, we summarize key studies regarding the structural basis for the S‐mediated cell entry of SARS‐CoV, MERS‐CoV and SARS‐CoV‐2. We also discuss the evolution of the S glycoprotein as a way to understand CoV cross‐species transmission from the viewpoint of structural biology. Finally, we provide an overview of the recent progress in therapeutic drugs targeting the SARS‐CoV‐2 S glycoprotein, including antibodies, nanobodies and peptide inhibitors.

## Overall structure of pre‐fusion CoV S glycoprotein

**Fig. 1:**
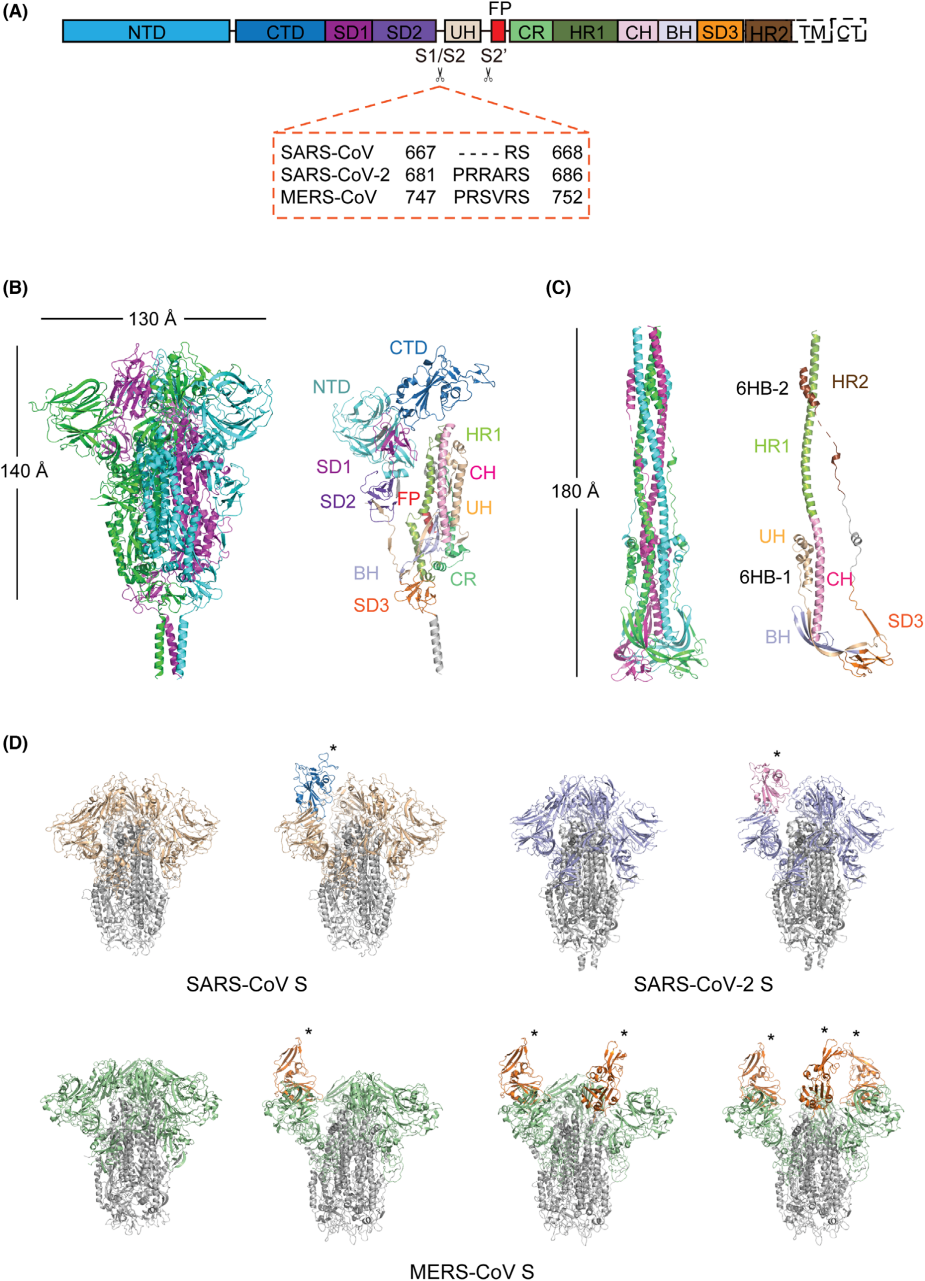
Overall structures of the S glycoprotein in the pre‐fusion and post‐fusion states. (A) Schematic representation of the structural domains in the S protomer. These domains are shown as boxes with the width reflecting the relative length of the amino acid sequence. The S1/S2 and S2’ cleavage sites are indicated by scissors. The S1/S2 cleavage sites of SARS‐CoV, SARS‐CoV‐2 and MERS‐CoV are shown in the dashed orange box. NTD, N‐terminal domain; CTD, C‐terminal domain; SD1, subdomain 1; SD2, subdomain 2; UH, upstream helix; FP, fusion peptide; CR, connecting region; HR1, heptad repeat 1; CH, central helix; BH, β‐hairpin; SD3, subdomain 3; TM, transmembrane domain; CT, cytoplasmic domain. (B) Overall structure of the SARS‐CoV‐2 S glycoprotein in the pre‐fusion state (PDB code: 6XR8). Left: Three protomers are shown as cartoon representations and coloured magenta, green and blue, respectively. Right: Structural domains of a protomer are coloured and labelled according to panel A. (C) Overall structure of the SARS‐CoV‐2 S glycoprotein in the post‐fusion state (PDB code: 6XRA). Left: Three protomers are shown as cartoon representations and coloured magenta, green and blue, respectively. Right: Structural domains of a protomer are coloured and labelled according to panel A. 6HB‐1, six‐helix bundle 1; 6HB‐2, six‐helix bundle 2. (D) Structural heterogeneity of SARS‐CoV, SARS‐CoV‐2 and MERS‐CoV S trimers. All S2 subunits are coloured grey. S1 subunits are coloured wheat for SARS‐CoV, light blue for SARS‐CoV‐2 and green for MERS‐CoV. ‘Up’ RBDs are indicated by asterisks and coloured blue for SARS‐CoV, pink for SARS‐CoV‐2 and orange for MERS‐CoV. SARS‐CoV S (PDB: 6ACC and 6ACD); SARS‐CoV‐2 S (PDB: 6VXX and 6VYB); MERS‐CoV S (PDB: 6Q05, 5X5F, 5X5C and 5X5C). [Colour figure can be viewed at wileyonlinelibrary.com]

S glycoprotein is a class I viral fusion protein [10] and is composed of an N‐terminal S1 subunit and a C‐terminal S2 subunit (Fig. 1A). The S1 subunit, which is mainly responsible for host cell receptor recognition, consists of an N‐terminal domain (NTD), C‐terminal domain (CTD) and two subdomains (SD1 and SD2) (Fig. 1A,B). The S2 subunit contains the elements for membrane fusion and is comprised of an upstream helix (UH), fusion peptide (FP), connecting region (CR), heptad repeat 1 (HR1), central helix (CH), β‐hairpin (BH), subdomain 3 (SD3), heptad repeat 2 (HR2), transmembrane domain (TM) and cytoplasmic tail (CT) (Fig. 1A,B). Two protease cleavage sites within the S glycoprotein – one at the boundary of the S1 and S2 subunits (S1/S2 site) and the other at the upstream of the FP (S2′ site) – are indispensable for cell entry [11, 12, 13] (Fig. 1A). The FP is a short, highly conserved segment of mainly hydrophobic residues that inserts into the target cell membrane to trigger the fusion event. Although the FP of SARS‐CoV was identified from extensive studies [14], the FP of SARS‐CoV‐2 is currently defined mainly from sequence comparison with SARS‐CoV and needs further experimental support.

On the viral surface, the protruding S glycoproteins form trimers (Fig. 1B), which undergo an irreversible, large‐scale transition from a metastable pre‐fusion state to a stable post‐fusion state for mediating membrane fusion. Cryo‐EM single‐particle analysis (SPA) has been pivotal in the structural determination of S trimers from different CoVs, including the pre‐fusion S trimers of SARS‐CoV [15, 16], SARS‐CoV‐2 [17, 18, 19] and MERS‐CoV [16, 20]. The overall structures of these CoV S trimers are similar, with three protomers interwoven together, resulting in a mushroom‐like shape ~ 140 Å in length and ~ 130 Å in width (Fig. 1B). Intact S proteins have also been observed on the SARS‐CoV‐2 virion by cryoelectron tomography (cryo‐ET) of chemically inactivated virus, with an average of 30–60 S trimers unevenly distributed across the envelope [21, 22, 23, 24]. The ectodomain of the S trimer is anchored to the viral envelope via a long flexible hinge, allowing the spike to adopt different orientations [21, 22, 23, 24]. The domains in the S1 subunit are mainly composed of β‐sheets, whereas the elements in the S2 subunit are mainly composed of helices (Fig. 1B). In the pre‐fusion state, the S trimer is in a metastable state, with the S1 subunits wrapping around the threefold axis and constraining the S2 fusion machinery (Fig. 1B). The pre‐fusion S trimers of SARS‐CoV, SARS‐CoV‐2 and MERS‐CoV display structural heterogeneity caused by the different conformations of the CTD, also known as the receptor‐binding domain (RBD). The pre‐fusion S trimers of SARS‐CoV, MERS‐CoV and HKU1 were reported to be stabilized by two consecutive proline substitutions (referred to as ‘2P’) in the loop between the HR1 and the CH [20]; this 2P strategy for pre‐fusion stabilization has been utilized in the development of SARS‐CoV‐2 mRNA vaccines [25].

Notably, the S1 and S2 subunits are both densely decorated with glycans. Sequence analysis identified 22, 23 and 23 potential N‐glycosylation sequons on the S protomers of SARS‐CoV‐2, SARS‐CoV and MERS‐CoV, respectively. Mass spectrometry analysis has experimentally verified 16 and 22 N‐glycosylation sites on the SARS‐CoV and MERS‐CoV S glycoproteins, respectively [26]. A recent study of the SARS‐CoV‐2 S glycoprotein detected a total of 22 N‐ and 17 O‐linked glycans, of which only the N234‐ and N709‐linked glycans are principally oligomannose type while the others are complex‐type glycans [27, 28]. The S1 subunit has 13 N‐ and 11 O‐linked glycans, whereas the S2 subunit has 9 N‐ and 6 O‐linked glycans [27, 28]. It has also been shown that most O‐linked glycans are located in close proximity to N‐linked glycans, revealing an ‘O‐follows‐N’ rule in the glycosylation pattern of the SARS‐CoV‐2 S protein [28].

## Overall structure of post‐fusion CoV S glycoprotein.

The pre‐ to post‐fusion transition of the S trimer is thought to be triggered by receptor binding and proteolytic processing, followed by the shedding of S1 subunits and the formation of the post‐fusion S2 trimer, which is structurally characterized by a six‐helix bundle containing the HR1 and HR2 repeats (Fig. 1C). Cryo‐ET studies of inactivated SARS‐CoV‐2 virions showed that the percentage of S trimers on the surface where S2 trimers are in the post‐fusion state varied from 0.1% to 74%, suggesting that post‐fusion S2 trimers can form spontaneously independent of later processing by target cells [19, 21, 22, 23, 24]. The wide range in the percent of post‐fusion S2 trimers observed is likely affected by the inactivation and purification methods used, indicating the instability of the S trimers on the envelope and providing clues for inactivated vaccine production. The role of post‐fusion S2 trimers on the virion surface remains under debate. It has been proposed that the post‐fusion S2 trimers may shield the pre‐fusion form from improper shedding or induce non‐neutralizing antibodies to evade the host immune system [19], but the observation of some virions harbouring only a small fraction of post‐fusion S2 trimers argues against this [22]. The high‐resolution structure of SARS‐CoV‐2 post‐fusion S2 trimer was determined by cryo‐EM SPA during the preparation of full‐length S glycoprotein under mild detergent conditions [19], while that of SARS‐CoV was derived from soluble S trimer processed by trypsin digestion, receptor binding and low pH treatment *in vitro* [29]. The structure of MERS‐CoV S glycoprotein in the post‐fusion state has yet to be reported. Consistent with the 91% amino acid sequence identity between the S2 subunits of SARS‐CoV‐2 and SARS‐CoV, the post‐fusion structures of SARS‐CoV‐2 and SARS‐CoV S2 homotrimers adopt a similar architecture with an overall root mean square deviation (RMSD) of 0.985 Å.

Compared to pre‐fusion S trimers, which exhibit different tilt angles on the virion surface, post‐fusion S2 trimers stand perpendicular to the envelope with relatively fixed orientations [21, 22]. The built model of the SARS‐CoV‐2 post‐fusion S2 trimer is a ~ 180‐Å long dumbbell, with a central helical bundle surrounded by short helices and β‐sheets at the distal end of the membrane [19] (Fig. 1C). The HR1 repeat flips over to form a continuous stem helix with the CH motif, extending the S2 segment ~ 80 Å longer and pointing the FP towards the target membrane. Three HR1‐CH long helices intertwine with each other to form the core region of the post‐fusion S2 trimer. The UH, BH and SD3 motifs are located around the C‐terminus of the central HR1‐CH helices, retaining their tertiary structures from the pre‐fusion state. The S1/S2‐S2’ fragment is non‐covalently associated with the post‐fusion structure despite cleavage at the S2’ site. A segment (residues 737 to 769) in the S1/S2‐S2’ fragment makes up three helical regions that pack against the groove of the CH motif to form the first six‐helix bundle structure (6HB‐1). The C‐terminal region of HR2 forms a short helix that constitutes the second six‐helix bundle structure (6HB‐2) with the HR1 coiled coil.

The post‐fusion S2 trimers remain highly glycosylated, with 8 out of 9 putative N‐linked glycans detected. Among them, five glycan sites (N1098, N1134, N1158, N1173 and N1194) are positioned along the long axis with regular spacing. These glycans mask the accessible surface of the corresponding regions during the transition from pre‐fusion to post‐fusion, which may protect the S2 subunit from antibody recognition.

## ‘Up’ RBD is required for CoV receptor binding

SARS‐CoV, SARS‐CoV‐2 and MERS‐CoV all utilize the CTD in the S1 subunit as a receptor‐binding domain (RBD) to specifically recognize their host cell receptors (i.e. ACE2 for SARS‐CoV and SARS‐CoV‐2 [1, 30, 31] and DPP4 for MERS‐CoV [32]). In a cryo‐EM study of the SARS‐CoV S trimer, we found different conformations of the RBD [15], indicating the positional flexibility of the RBD relative to other parts of the S trimer (Fig. 1D). More importantly, structural analysis showed that the ACE2‐binding sites on the RBD are buried in the closed form of the S trimer, with all three RBDs in a ‘down’ conformation. The ACE2‐binding site is only fully exposed when one RBD adopts an ‘up’ conformation by protruding from the S trimer. In a cryo‐EM study of the SARS‐CoV S trimer complexed with ACE2, we showed that ACE2 could only bind ‘up’ RBD [33]. Therefore, we proposed and then demonstrated that the ‘down’ to ‘up’ conformational change of at least one RBD is a prerequisite for receptor binding of SARS‐CoV. These conclusions were subsequently supported by similar observations during the structural determination of the MERS‐CoV [16, 20] and SARS‐CoV‐2 [17, 18, 34] S trimers.

The S trimers of most human coronaviruses, including HCoV‐229E [35, 36], HCoV‐OC43 [37], HCoV‐NL63 [38, 39] and HKU1 [40], have only exhibited a closed conformation in cryo‐EM structural studies. However, for SARS‐CoV, MERS‐CoV and SARS‐CoV‐2, an open form of the S trimer with ‘up’ RBD has been observed. Additionally, an intermediate state of SARS‐CoV‐2 S trimer that is between the closed and open form and in which one RBD has weak density was also recently reported [24, 34]. S trimer with one ‘up’ RBD has been observed in SARS‐CoV, while one to three ‘up’ RBDs have been captured in MERS‐CoV (Fig. 1D). As for SARS‐CoV‐2, wild‐type S trimer mainly adopts a one‐RBD‐up conformation; S trimers with a two‐RBD‐up conformation have been principally obtained in SARS‐CoV‐2 variants [41, 42, 43]. Moreover, the SARS‐CoV‐2 S trimer incubated with ACE2 revealed a two‐RBD‐up conformation, indicating that ACE2 binding could lead to a more open trimer conformation [34]. In Fig. 1D, we illustrate the structural heterogeneity of wild‐type SARS‐CoV, SARS‐CoV‐2 and MERS‐CoV S trimers. The molecular mechanisms underlying these different RBD conformations in SARS‐CoV, SARS‐CoV‐2 and MERS‐CoV S trimers remain to be elucidated.

A linoleic acid (LA) binding pocket was recently identified in the SARS‐CoV‐2 S trimer [44]. A similar pocket with bound LA was also identified in the S trimer of the pangolin coronavirus PCoV_GX, which is closely related to SARS‐CoV‐2 [45]. This pocket could be of interest in drug development as ligands bound in this pocket could lock the RBD in the ‘down’ conformation and thus prevent binding to ACE2. Although this pocket has not been experimentally verified in the SARS‐CoV S glycoprotein, it is predicted to exist as the amino acid residues in this region are highly conserved with those found in SARS‐CoV‐2.

## Interaction between CoV S glycoprotein and host receptor

**Fig. 2:**
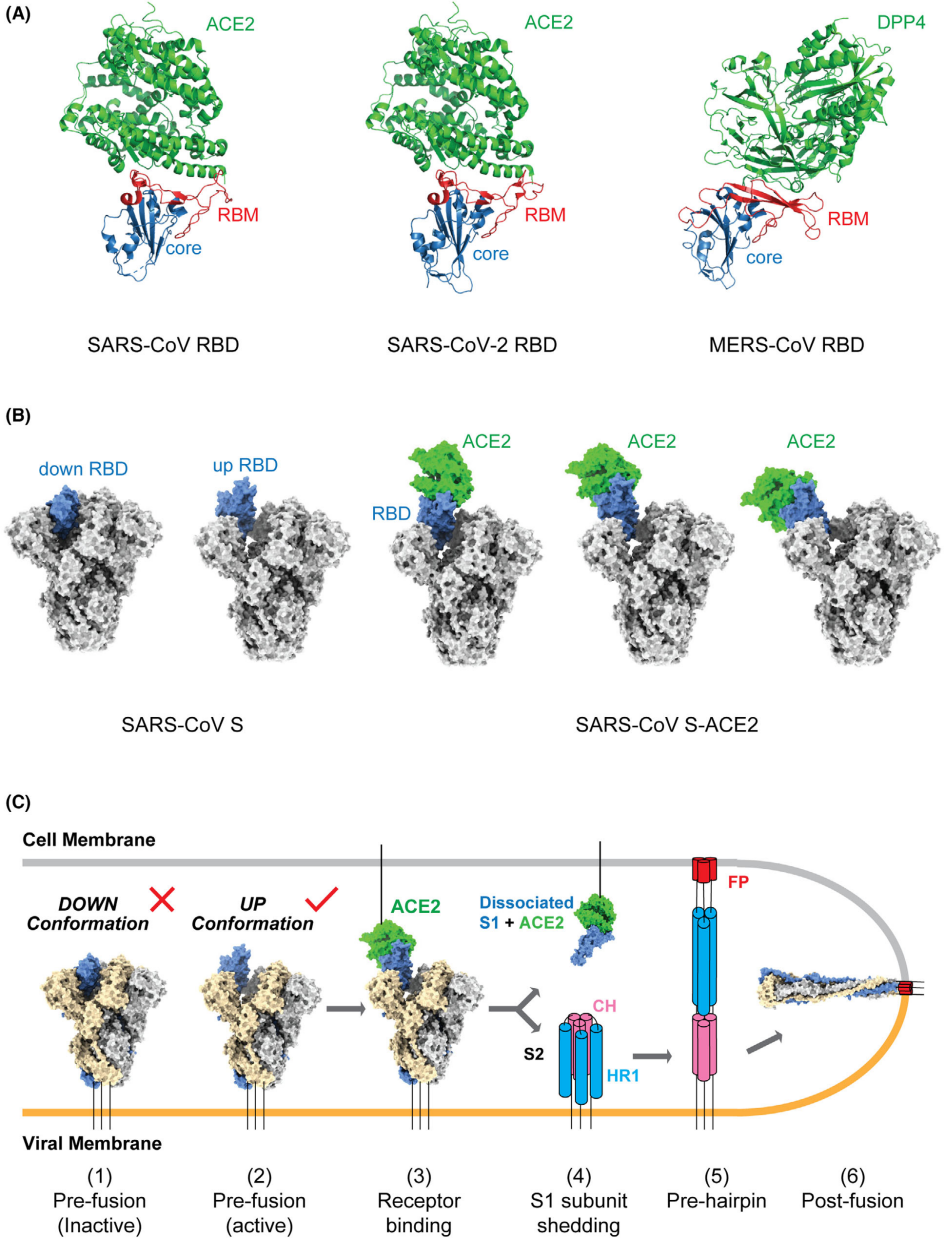
Interaction between CoV S glycoprotein and host receptor. (A) Overall structures of the SARS‐CoV (left) and SARS‐CoV‐2 RBDs (middle) bound to ACE2 and MERS‐CoV RBD (right) bound to DPP4. RBD core domains are coloured blue and RBMs are coloured red. Both the ACE2 and DPP4 receptors are coloured green. PDB codes: SARS‐CoV RBD–ACE2 complex, 2AJF; SARS‐CoV RBD‐2–ACE2 complex, 6M0J; MERS‐CoV RBD–DPP4 complex, 4L72. (B) Overall structures of SARS‐CoV S glycoprotein (left) and ACE2‐bound SARS‐CoV S glycoprotein (right). S glycoprotein is shown in grey and the dynamic RBD in blue. ACE2 is coloured green. PDB codes: 6ACG, 6ACJ, 6ACK, 6ACD and 6ACC. (C) Model of the pre‐fusion to post‐fusion transition of the SARS‐CoV S trimer. The down‐to‐up conformational transition of RBD (1–2) exposes the receptor‐binding site, followed by ACE2 binding (3), ‘opening’ up of the S trimer, shedding of the S1 subunits (4), and a large‐scale conformational change of the unwrapped S2 subunits for FP insertion into the cell membrane (5, pre‐hairpin state) and the formation of a six‐helix bundle to enable membrane fusion (6). This model is based on current knowledge and could be optimized with future data. [Colour figure can be viewed at wileyonlinelibrary.com]

Prior to cryo‐EM SPA, X‐ray crystallography was the major method utilized to study the interaction between CoV RBD and host receptors at the atomic level. Crystal structures of SARS‐CoV, SARS‐CoV‐2 and MERS‐CoV RBDs bound to their respective receptors have all been determined [46, 47, 48, 49, 50], providing molecular details about RBD‐receptor interactions (Fig. 2A). The RBDs of these three CoVs have a similar architecture, consisting of a β‐sheet core and an inserted loop known as the receptor‐binding motif (RBM) (Fig. 2A). The core has a twisted five‐stranded antiparallel β sheet with short connecting helices that stabilize the RBD structure, and the RBM contains two short strands and loops that directly interact with ACE2 (SARS‐CoV, SARS‐CoV‐2) or DPP4 (MERS‐CoV) (Fig. 2A).

The SARS‐CoV and SARS‐CoV‐2 RBDs bind ACE2 in a very similar manner (Fig. 2A). In structures of both complexes, the extended RBM contacts the bottom side of the small lobe of ACE2. The buried surface area, hydrophilic interaction network and the number of interacting residues are all highly similar between these two RBD/ACE2 interfaces. The cradling of the N‐terminal helix of ACE2 by the outer surface of the RBM results in a large buried surface of 1687 and 1699 Å^2^ at the SARS‐CoV‐2 and SARS‐CoV RBD/ACE2 interfaces, respectively. Both interfaces contain a network of hydrophilic interactions, with 13 hydrogen bonds and 2 salt bridges at the SARS‐CoV‐2 RBD/ACE2 interface, and 13 hydrogen bonds and 3 salt bridges at the SARS‐CoV RBD/ACE2 interface. With a distance cut‐off of 4 Å, a total of 17 residues of the SARS‐CoV‐2 RBD are in contact with 20 residues of ACE2 while 16 residues of the SARS‐CoV RBD are reported to bind to 20 residues of ACE2. Seventeen of the 20 ACE2 residues that interact with the two different RBDs are the same and most of the contacting residues are located at the N‐terminal helix.

In addition to those different states observed for the RBD, other conformational states of SARS‐CoV S have been observed during the binding process, including three conformational states of the S‐ACE2 complex; the dissociated S1‐ACE2 complex and the S2 trimer in the post‐fusion state [33]. In the three S‐ACE2 complex states, the observation of increased ‘up’ angles of the RBD upon ACE2 binding provided a clue that receptor engagement helps to further ‘open’ the S trimer (Fig. 2B). Therefore, besides being an important interaction for viral attachment to host cells, ACE2 binding also facilitates shedding of the S1 subunits and exposure of the S2 subunits essential for membrane fusion. A cryo‐EM study of SARS‐CoV‐2 has also resolved ten distinct conformations of SARS‐CoV‐2 S and S‐ACE2 complexes, including tightly closed and unbound trimers, open trimers, open trimers with bound ACE2 and dissociated monomeric S1‐ACE2 complex [34]. Although it was found that one SARS‐CoV‐2 S trimer was able to bind to up to three ACE2 molecules [34], the exact number of bound ACE2 required for subsequent state transition of the S trimer is still unknown. Based on these results, we can establish a relatively simple model to illustrate the likely pre‐fusion to post‐fusion transition of the SARS‐CoV and SARS‐CoV‐2 S trimers during cell entry (Fig. 2C). The process begins with a down‐to‐up conformational change of RBD to expose the receptor‐binding site (receptor‐binding inactive to active), followed by ACE2 binding, opening up of the S trimer, shedding of the S1 subunits, a large‐scale conformational change of the unwrapped S2 subunits for FP insertion into the cell membrane and finally the formation of a six‐helix bundle to enable membrane fusion (Fig. 2C). However, several key questions are still not answered for this model. For example, we do not know whether the binding of ACE2 to one ‘up’ RBD is sufficient to induce S1 shedding or if binding of three ACE2 molecules to all three RBDs is required. We also do not know how the S2 trimers transition to form the six‐helix bundle at the atomic level. Future studies are needed to resolve these questions and to optimize our proposed model.

## Roles of proteases in CoV entry

In addition to receptor binding, protease cleavage also plays a critical role in CoV entry. As described above, CoV S glycoprotein contains two proteolytic cleavage sites, S1/S2 and S2′. The S1/S2 site is located at the junction of the S1 and S2 subunit, and the S2′ site is upstream of the FP within the S2 subunit. Previous studies have shown that trypsin cleavage of SARS‐CoV S occurs sequentially, with the S1/S2 cleavage occurring first and enhancing subsequent cleavage at the S2′ site. It is thought that the second cleavage event at the S2′ site is crucial for fusion activation of the S glycoprotein [51]. A recent study also supported that the S2′, but not the S1/S2, cleavage site is indispensable for SARS‐CoV‐2 S to mediate syncytia formation and infection [52]. ACE2 engagement strongly enhances SARS‐CoV‐2 S2′ cleavage, suggesting that ACE2 binding likely facilitates exposure of the S2′ site [52, 53]. Protease processing can occur in different processes such as during S biogenesis, after releasing into the extracellular fluid, during cell entry at the cell membrane or in endosomes, modulating the route of viral entry and affecting cell and tissue tropism [11, 12, 13].

The S trimers of SARS‐CoV‐2 and MERS‐CoV are partially cleaved and form non‐covalently associated S1/S2 trimers in contrast to the uncleaved S trimers observed for SARS‐CoV and all other known sarbecovirus S trimers [16, 18, 24, 31, 34, 54]. One important reason for this is the presence of four amino acid residues, P‐R‐R‐A and R‐S‐V‐R, at the S1/S2 junction in SARS‐CoV‐2 and MERS‐CoV, respectively. This insertion generates a polybasic cleavage site, which enables effective cleavage by furin, a ubiquitously expressed membrane‐bound protease, during spike biosynthesis [55, 56, 57, 58, 59]. Furin activation of SARS‐CoV‐2 S at the S2′ position has also been observed, but with less efficiency than that at the S1/S2 site [53]. A structural study revealed that furin cleavage could help induce the ‘open’ conformation of the SARS‐CoV‐2 S protein [34]. Additionally, furin cleavage at the S1/S2 site was found to be essential for SARS‐CoV‐2 entry into human lung cells [59]. SARS‐CoV‐2 virus lacking the furin site showed reduced viral titres in infected ferrets and lost its ability to transmit to cohoused sentinel animals [60]. SARS‐CoV‐2 with mutations in the furin cleavage site also demonstrated attenuated pathogenesis in hamster and mouse models [61]. The mutation P681R found in the SARS‐CoV‐2 delta variant was reported to facilitate cleavage of the S glycoprotein and enhance viral fusogenicity [62]. Thus, the S glycoproteins of SARS‐CoV‐2 and MERS‐CoV are more readily primed for membrane fusion, which was also proposed for the dynamic conformation sampling of the S glycoprotein, high viral infectivity and expanded viral tropism of these two viruses.

Besides furin and furin‐like proteases, other proteases are thought to be involved in the CoV entry process [31, 58, 63, 64, 65, 66, 67, 68], such as trypsin, transmembrane serine protease 2 (TMPRSS2) and cathepsins. Cleavage by trypsin and TMPRSS family members usually occurs in the extracellular space and at the cell surface, respectively, while cleavage by cathepsins is usually in endosomes. Protease availability determines the entry routes of CoVs, either via an ‘early pathway’ at the plasma membrane or a ‘late pathway’ at the endosomal membrane [12]. For SARS‐CoV‐2, the furin‐cleaved S protein requires further processing at the S2′ site to be fully activated. The S2′ site can be processed by TMPRSS2 at the cell surface, triggering the early fusion pathway [31, 53, 58]. TMPRSS2 is expressed in many epithelial cells and has been studied as an antiviral target to inhibit SARS‐CoV‐2 entry [31]. Alternatively, in the absence of exogenous or membrane‐bound proteases, ACE2‐bound SARS‐CoV‐2 can also be internalized via clathrin‐mediated endocytosis [69]. The low pH environment within the endosome activates cathepsin L cleavage at the S2′ site, triggering the late fusion pathway [31, 58]. SARS‐CoV‐2 is more dependent on TMPRSS2 than SARS‐CoV during entry, largely due to the furin site in the SARS‐CoV‐2 S protein [70]. A combination of inhibitors of endosomal acidification inhibitor and TMPRSS2 has proved more potent in preventing SARS‐CoV‐2 infection [70].

Although furin, TMPRSS2 and cathepsin L have been most extensively studied in the proteolytic activation process of SARS‐CoV‐2, trypsin, TMPRSS4, TMPRSS11A, TMPRSS11D and TMPRSS11E have also been shown to enhance SARS‐CoV‐2 S‐mediated cell–cell fusion [63]. The broad and redundant protease usage by SARS‐CoV‐2 may contribute to its infectivity and transmissibility among humans and other species. These proteases and their working mechanisms require more comprehensive studies, which could offer further insights into SARS‐CoV‐2 entry and provide more potential targets for antiviral drug development.

## Structural insights into the evolution of the CoV S glycoprotein

**Fig. 3:**
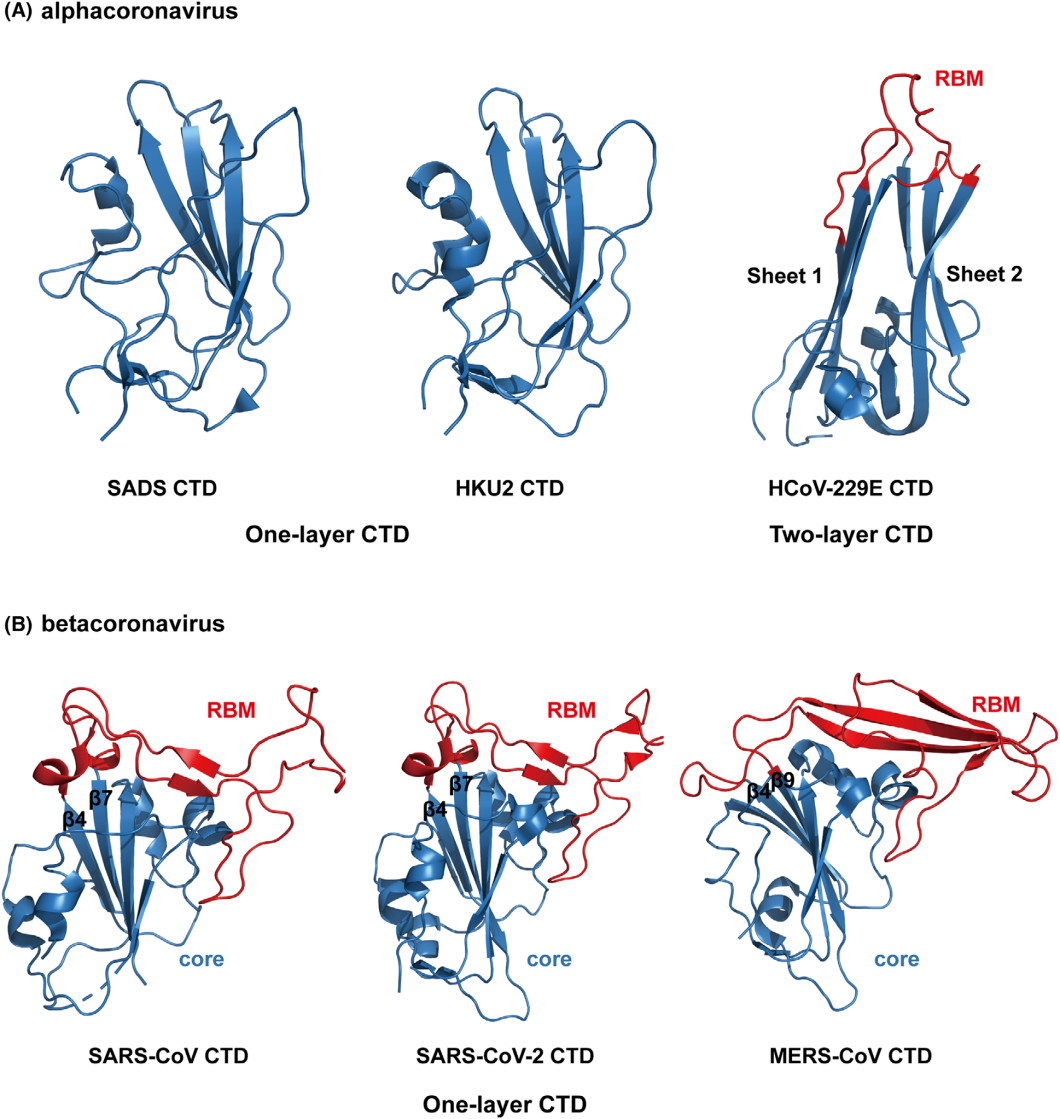
Structural comparison of CTDs from alpha‐ and betacoronaviruses. (A) *Alphacoronavirus* SADS, HKU‐2 and HcoV‐229E CTD structures. The CTDs of SADS and HKU‐2 belong to the one‐layer CTD subtype while that of HcoV‐229E belongs to the two‐layer CTD subtype. The CTD core is shown in blue and RBM in red. PDB codes: SADS, 6M16; HKU‐2, 6M15; HcoV‐229E, 6U7H. (B) *Betacoronavirus* SARS‐CoV, SARS‐CoV‐2 and MERS‐CoV CTD structures. These three *Betacoronavirus* CTDs belong to the one‐layer CTD subtype. The CTD core is shown in blue and RBM in red. PDB codes: SARS‐CoV, 2AJF; SARS‐CoV‐2, 6M0J; MERS‐CoV, 4L7A. [Colour figure can be viewed at wileyonlinelibrary.com]

Previous studies have revealed the structural basis for the evolution of the S glycoprotein, especially the RBD for receptor interaction [71]. Under selective pressure, recombination and mutations in the RBD have enabled adaptation to receptors of new hosts [71]. For instance, SARS‐CoV is considered to have originated in bats and was transmitted to humans through palm civets in markets. SARS‐CoV RBD binds to human ACE2 (hACE2) more tightly than civet SARS‐CoV RBD. A structural study found two key mutations in the SARS‐CoV RBD, K479N and S487T, that allowed for civet‐to‐human transmission [72]. Recently, the structures of the S proteins from the alphacoronaviruses porcine SADS‐CoV and closely related bat HKU‐2 were reported by us and other groups [73, 74]. One interesting finding was that the CTDs of SADS and HKU‐2 adopt a one‐layer β‐sheet structure [73] (Fig. 3A). This architecture is different from the CTD structures of other alphacoronaviruses, including HCoV‐229E [36] (Fig. 3A), but is similar to the core subdomain structures of betacoronaviruses like SARS‐CoV, SARS‐CoV‐2 and MERS‐CoV [46, 49, 50] (Fig. 3B). The protein receptor for SADS has not been identified, but we think that its CTD would not function as a typical RBD due to the absence of an RBM (Fig. 3A). In contrast, the RBM between the β4 and β7 strands in SARS‐CoV and SARS‐CoV‐2 and between the β4 and β9 strands in MERS‐CoV is thought to have arisen through recombination with other CoVs and along with additional mutations, resulting in the ability of these viruses to efficiently bind their respective receptors [46, 49, 50] (Fig. 3B).

Currently, it is still unclear how and when SARS‐CoV‐2 originated and crossed the species barrier to infect humans. Several bat CoVs closely related to SARS‐CoV‐2 have been reported, indicating an origin in bats is likely. Such bat CoVs include RaTG13 found in Yunnan province, China with 96.2% sequence identity [1] and BANAL‐52, BANAL‐103 and BANAL‐236 found in North Laos with > 95% sequence identity with the SARS‐CoV‐2 genome [75]. BANAL‐52 has 96.8% sequence identity with SARS‐CoV‐2, and more importantly, the RBDs of BANAL‐52, BANAL‐103 and BANAL‐236 differ from the SARS‐CoV‐2 RBD by only one or two residues, resulting in efficient binding to human ACE2 and pseudovirus entry mediated by human ACE2 [75, 76]. However, speculation that more than 20 years of sequence evolution would have been needed to fulfil the divergence between SARS‐CoV‐2 and bat CoVs suggests that these bat CoVs are not the direct progenitors of SARS‐CoV‐2 [77]. Several CoVs related to SARS‐CoV‐2 have also been identified in smuggled Malayan pangolins (*Manis javanica*) in China's Guangxi (GX) and Guangdong (GD) provinces. Analysis of these pangolin CoV (PCoV) genomes found 85.5%–92.4% sequence identity with the SARS‐CoV‐2 genome [78, 79, 80, 81]. However, as infected pangolins did show clinical signs of infection, they are unlikely to be the reservoir of SARS‐CoV‐2 and more likely were infected after spillover from natural hosts [80].

**Fig. 4:**
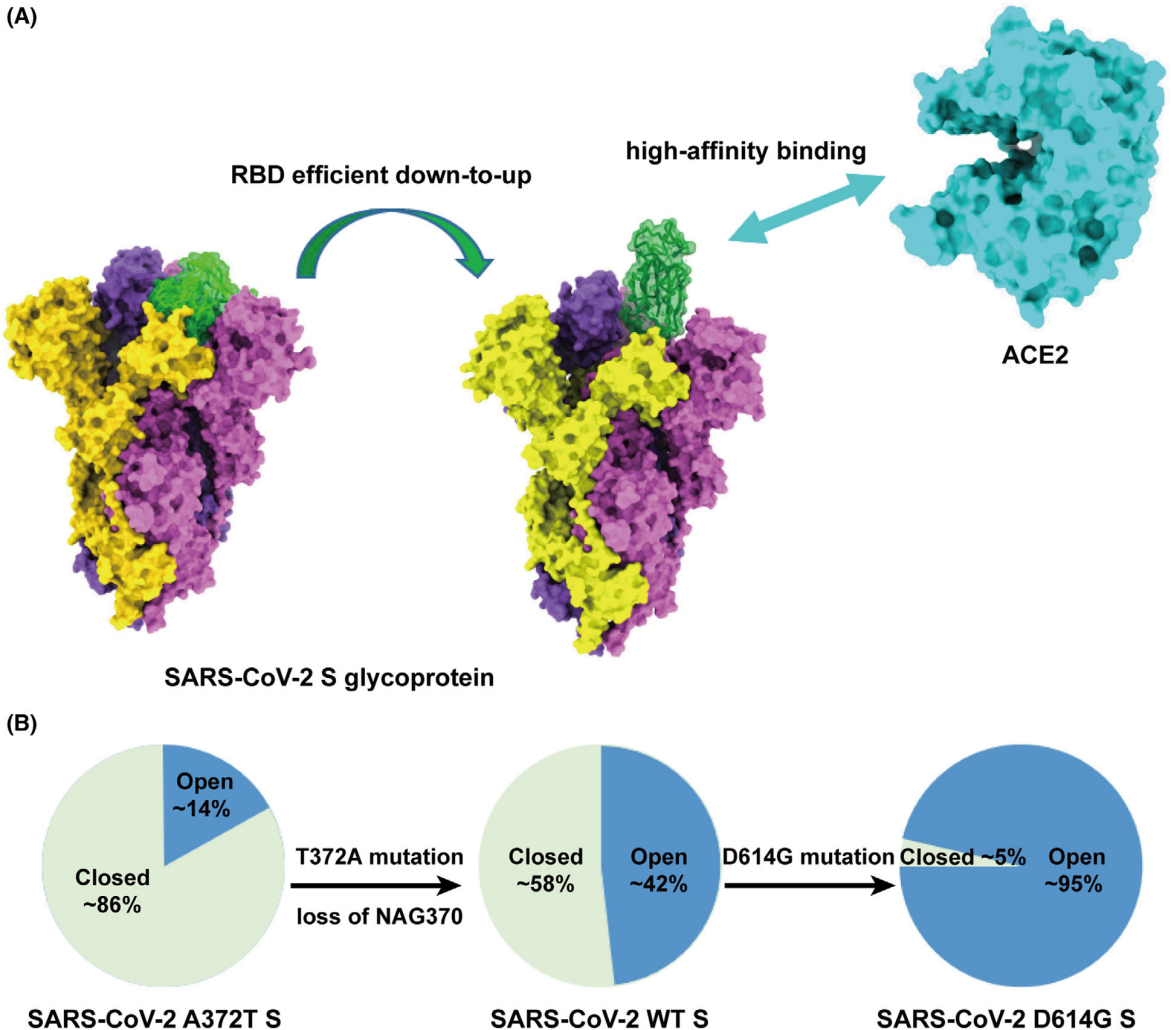
RBD efficient down‐to‐up conformational change and high‐affinity binding to ACE2 are both required for SARS‐CoV‐2 to gain high infectivity. (A) The structures of SARS‐CoV‐2 S glycoprotein and human ACE2 are shown in surface representation. PDB codes: SARS‐CoV‐2 S glycoprotein, 6VXX, 6VYB; human ACE2, 6M0J. (B) The percentage of close and open RBD conformations for SARS‐CoV‐2 A372T, wild‐type (WT) and D614G S glycoprotein. Open conformation is shown in blue and closed conformation in green. [Colour figure can be viewed at wileyonlinelibrary.com]

We and others recently reported the cryo‐EM structures of the RaTG13, PCoV_GD and PCoV_GX S glycoproteins [34, 45, 82]. These S glycoproteins, including the RBD, are structurally similar to that of SARS‐CoV. One significant difference is that no ‘open’ form S trimer was observed in the cryo‐EM studies of these three CoVs [34, 45, 82]. Binding and pseudovirus entry experiments revealed that RaTG13 pseudovirus was not able to efficiently enter cells expressing human ACE2, and the binding of its RBD to ACE2 was very weak [45]. PCoV_GX pseudovirus could enter cells expressing human ACE2, but the efficiency was also lower than that of the SARS‐CoV‐2 pseudovirus even though its RBD exhibited an ACE2‐binding ability nearly the same as that of the SARS‐CoV‐2 RBD [45]. These results led us to propose that an efficient down‐to‐up conformational change of the RBD and high‐affinity binding of the RBD to human ACE2 are both required for SARS‐CoV‐2 to have high infectivity (Fig. 4A). We also used sequence comparison and mutagenesis to pinpoint several key sites in the SARS‐CoV‐2 RBD, including Y449, N501 and Y505, that contribute to tight binding to hACE2 [45].

Furthermore, we and others recently found that loss of N370 glycosylation is an important evolutionary event that enhanced the infectivity of SARS‐CoV‐2 [83, 84]. The N‐glycosylation ‐NXS/T‐ sequon is highly conserved among sarbecoviruses at the N370 site, and the N370‐linked glycans have also been observed in the cryo‐EM structures of RaTG13, PCoV_GD and PCoV_GX S glycoproteins. However, in the SARS‐CoV‐2 S glycoprotein, the sequon is instead ‐NSA‐, resulting in the loss of the N370 glycosylation. SARS‐CoV‐2 pseudovirus with a mutation restoring the N370 glycosylation site (A372S) exhibited significantly reduced cell entry capacity compared to wild‐type. Conversely, an S372A mutation to disrupt the N370 glycosylation site in RaTG13 pseudovirus resulted in significantly enhanced cell entry capacity compared to wild type. Structural studies further showed that, compared to wild‐type SARS‐CoV‐2 S, loss of N370 glycosylation increased the percentage of ‘open’ form SARS‐CoV‐2 S trimers among all particles from ~ 14% to ~ 42%, which is expected to facilitate binding to ACE2 receptor [18, 83] (Fig. 4B). Future studies are still needed to completely reveal the molecular basis for the evolution of SARS‐CoV‐2 S glycoprotein, especially regarding the efficient RBD down‐to‐up conformational change essential for receptor binding.

## Diversity of SARS‐CoV‐2 mutations

The mutation rate of CoV genomes is ~ 10^−4^ substitutions per site per year [85]. With the global spread of SARS‐CoV‐2, it is no surprise that mutations emerge continuously. In January 2020, the first notable genome mutation, A23403G, appeared and became dominant all over the world, resulting in a D614G substitution in the S glycoprotein [86, 87]. The SARS‐CoV‐2 variant carrying this mutation showed increased viral loads in the upper respiratory tract of infected individuals, enhanced viral replication in human lung epithelial cells and primary respiratory tissues, and greater transmission ability and infectivity [88]. Structural determination with SPA found that there was a significant increase, from ~ 47% to ~ 95%, in the percent of ‘open’ form S trimers with this D614G mutation [89] (Fig. 4B).

Five SARS‐CoV‐2 mutants with the D614G substitution have raised great concern due to their increased transmission ability, virulence, antibody resistance and/or expansion of host range. These mutants, which were defined as variants of concern (VOCs), include Alpha (B.1.1.7), Beta (B.1.351), Gamma (P.1), Delta (B.1.617.2) and Omicron (B.1.1.529). In these VOCs, mutations in the RBD either directly affect receptor binding and/or allow escape from antibody neutralization. One notable example is the N501Y mutation, which appears in the Alpha, Beta, Gamma and Omicron variants and enhances ACE2‐binding affinity by ~ 10‐fold [90, 91, 92]. The K417N/K417A is another common mutation in these VOCs. Although the K417N/K417A substitution is thought to decrease binding to ACE2, it is also a hot‐spot mutation site for escaping antibody neutralization. Several other mutations found in these VOCs, including N440K, L452R and T478K, are not found at the RBD/ACE2 interface and are thought to mainly affect binding and neutralization of antibodies [93, 94].

In addition to human ACE2, SARS‐CoV‐2 S/RBD binds to ACE2 from other animal species. Rhesus macaques, dogs, cats, cattle, hamsters, ferrets and minks are all susceptible hosts [95, 96, 97, 98, 99, 100], indicating SARS‐CoV‐2 has a broad host range. Natural SARS‐CoV‐2 infections have been reported in dogs, cats, tigers, lions and minks [101]. These findings highlight the importance of surveillance efforts to prevent future outbreaks. SARS‐CoV‐2 variant ‘cluster V’ (B.1.1.298) was identified in mink in the Netherlands with four mutations in the spike protein (Y453F, I692V, M1229I and deletion of amino acids 69–70) [102, 103]. A cryo‐EM study of this variant's S protein found closed, one RBD ‘up’ and two RBD ‘up’ states [43]. Structural analysis indicated that the Y453F substitution in the RBD is a species‐specific adaptive mutation, increasing binding to mink ACE2 [43, 104]. This mutated virus strain circulated among the farmed minks, eventually leading to the first known animal‐to‐human transmission of SARS‐CoV‐2 [105].

## Neutralizing antibodies targeting CoV S glycoprotein

Infection with the highly pathogenic SARS‐CoV, MERS‐CoV or SARS‐CoV‐2 viruses stimulates rapid antibody production in the sera, including neutralizing antibodies that bind to the S glycoprotein. Convalescent plasma therapy has proven effective in treating patients infected by SARS‐CoV and MERS‐CoV [106, 107, 108, 109]. Limited clinical data available for SARS‐CoV‐2 also suggested that this therapy was effective in reducing viral load and improving the survival of infected individuals [110, 111, 112, 113]. As a result, convalescent plasma therapy was issued an emergency use authorization (EUA) and employed in China during the early stages of COVID‐19. However, the significant side effects and limited efficacy of convalescent plasma underscored the need for isolating monoclonal neutralizing antibodies specifically targeting the SARS‐CoV‐2 S glycoprotein. A large number of such antibodies have been isolated from convalescent patient samples, immunized animals and phage display libraries [114, 115, 116, 117]. Their binding and neutralization activities have been extensively studied, and some of the most potent ones were studied in clinical trials or approved for clinical use by regulatory authorities. Representative ones include REGN10933/REGN10987 from Regeneron, LY‐CoV555/LY‐CoV016 from Eli Lily, AZD1061/AZD8895 from AstraZeneca, BRII‐196/BRII‐198 from Brii and VIR7831 from GlaxoSmithKline.

**Fig. 5:**
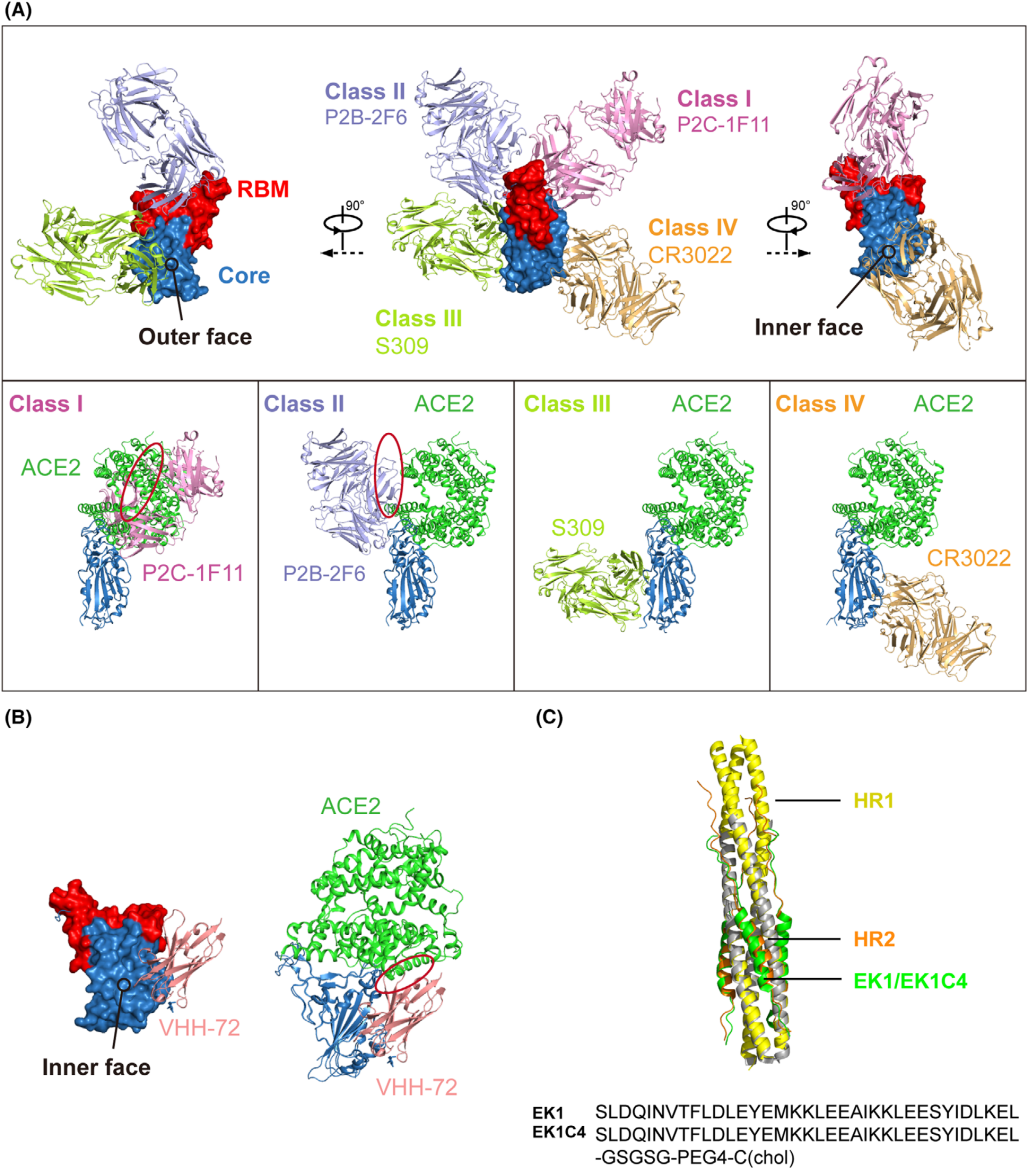
Inhibitors of SARS‐CoV‐2 S glycoprotein. (A) Anti‐RBD neutralizing antibodies. Upper panel: Antibodies are classified into four classes based on different epitopes. P2C‐1F11, P2B‐2F6, S309 and CR3022 are shown to represent Class I, Class II, Class III and Class IV, respectively. RBD is shown in surface representation, with RBM red and core domain blue. The inner and outer faces are indicated. Lower panel: Each antibody from the upper panel is separately shown superimposed onto the SARS‐CoV‐2 RBD‐ACE2 complex. The circle indicates the clash with ACE2. (B) The nanobody VHH‐72 binding the core domain of the SARS‐CoV‐2 RBD. The circle indicates the clash with ACE2. (C) 6HB‐targeting peptides EK1 and EK1C4. HR1 is shown in yellow, HR2 in orange and EK1/EK1C4 in green. [Colour figure can be viewed at wileyonlinelibrary.com]

The NTD, RBD and S2 subunit of CoV S glycoprotein are all susceptible regions for antibody binding, with RBD the predominant target. The SARS‐CoV‐2 RBD‐directed antibodies were initially grouped into four major classes based on different binding epitopes. Class I and Class II antibodies bind at the RBM region, whereas Class III and Class IV antibodies bind at the core domain [118] (Fig. 5A). With more binding and epitope data, the epitopes of RBD‐directed antibodies have been more finely mapped as seven communities (RBD 1–7) [119] or six classes (class 1–6) [120]. For these groupings of RBD‐directed antibodies, structural determination of numerous antibody‐S/RBD complexes has provided important knowledge about these epitopes at the atomic level. Here, we briefly summarize the representative members of the Class I–IV antibodies from a structural perspective.

P2C‐1F11 is a member of Class I that we previously isolated and characterized [114]. The crystal structure of the SARS‐CoV‐2 RBD‐P2C‐1F11 complex showed that P2C‐1F11 binds on the ridge of RBM with a buried surface area of ~ 955 A^2^ that largely overlaps with the ACE2‐binding site (Fig. 5A). The binding epitope of P2C‐1F11 on RBD shares as many as 11 residues with that of ACE2 (K417, Y453, L455, F456, A475, F486, N487, Y489, Q493, G502 and Y505). Therefore, P2C‐1F11 strongly competes with ACE2 for binding, which is expected to be critical for highly potent neutralization against SARS‐CoV‐2 [121].

The binding angle of the Class II antibody P2B‐2F6 is tilted from that of P2C‐1F11 with less steric hindrance of ACE2 [114] (Fig. 5A). Correspondingly, based on the structural alignment, only G446 and Y449 on the SARS‐CoV‐2 RBD were predicted to overlap between the P2C‐2F6 epitope and the ACE2‐binding site [121]. The Class III antibody S309 was isolated from the memory B cells of a SARS‐CoV convalescent patient and exhibited cross‐neutralizing activity against SARS‐CoV‐2 [122]. Structural information showed that S309 binds to the outer surface of the core subdomain, which is far away from the ACE2‐binding site on the RBM (Fig. 5A). The binding epitope of S309 on both the SARS‐CoV and SARS‐CoV‐2 S glycoproteins is highly conserved, with 17 out of 22 residues being identical, including the region Y369‐F392 and the N343‐linked glycans [122]. The representative Class IV antibody CR3022 was also derived from a patient who recovered from SARS‐CoV infection and binds to the inner surface of the core subdomain (Fig. 5A). Similar to S309, the epitope of CR3022 is also highly conserved between SARS‐CoV and SARS‐CoV‐2, with 23 of the 26 interacting residues identical [123].

In summary, the most potent neutralizing antibodies are largely from Class I and Class II as they directly interfere with ACE2 binding. Class III and IV antibodies have little or no competition with ACE2 and thus generally have lower neutralizing activity. However, Class III and IV antibodies tend to exhibit a broad cross‐reactivity because of their conserved epitopes among SARS‐CoV, SARS‐CoV‐2 and other sarbecoviruses. Class I and Class II antibodies are more prevalent than Class III and IV, suggesting that the RBM is more immunogenic and vulnerable compared to other regions of the RBD.

## Nanobodies

Nanobodies (single‐domain antibodies) isolated from camelids or recombinant libraries also display therapeutic promise for COVID‐19. One major advantage of nanobodies is their smaller size, which allows them to bind to regions inaccessible to conventional antibodies. Throughout the COVID‐19 pandemic, large numbers of nanobodies against SARS‐CoV‐2 RBD have been isolated and characterized [116, 124, 125, 126]. Here, we will discuss one of the first reported neutralizing nanobodies, VHH‐72, as an example. VHH‐72 was isolated from a llama immunized with SARS‐CoV and was subsequently shown to cross‐neutralize SARS‐CoV‐2 and circulating SARS‐CoV‐2 variants *in vitro* [127]. Additionally, VHH‐72 could decrease SARS‐CoV‐2 replication in prophylactic and therapeutic settings in both mice and hamsters [128]. Further study has indicated that the main epitope of VHH‐72 is found on the core subdomain with small clashes with ACE2 (Fig. 5B), which makes it a highly potent cross‐neutralizer [125]. ExeVir Bio did begin a clinical trial to evaluate XVR011 (VHH‐72‐Fc) in patients hospitalized with SARS‐CoV‐2 infection in September 2021, but generally, clinical research of nanobodies for treating COVID‐19 patients lags behind conventional antibodies.

## The challenge of viral mutations that escape antibody neutralization

The five current SARS‐CoV‐2 VOCs generally contain ~ 10–40 site mutations in the whole genome, with ~ 10 mutations in the S glycoprotein, except for Omicron, which has > 30. The effect of these mutations has been extensively studied [129, 130, 131, 132, 133, 134, 135]. In addition to their effect on receptor engagement as described above, these mutations can allow for escape from neutralizing antibodies and vaccines. Mutations at K417 and E484 in the Beta (B.1.351), Gamma (P.1) and Omicron (B.1.1.529) variants are mainly resistant to neutralizing antibodies from Class I and Class II, respectively [136, 137, 138, 139]. Some Class IV antibodies show a significant loss of neutralization potency against the triple mutation S371L/S373P/S375F found in Omicron [140, 141]. The L452R and T478K mutations reported in Delta (B.1.617.2) allow for evasion from some Class III antibodies [93, 94]; Antibodies of the same class can respond differently to mutations due to different binding microenvironments. For example, the Class I antibody P2C‐1F11 is effective against Beta and Gamma even though most Class I antibodies are not [137, 138]. Omicron is a notable variant with as many as 15 mutations in the RBD, which leads to resistance to most antibodies from Classes I and II and some from Classes III and IV [140, 141, 142].

Accordingly, the emerging variants, especially Beta, Gamma and Omicron, have heavily affected the development and use of antibody therapies. For example, Beta, Gamma and Omicron have all escaped the therapeutic cocktail of LY‐CoV555/LY‐CoV016 while REGN10933/REGN10987 and AZD1061/AZD8895 have been effective against Alpha, Beta, Gamma and Delta but not Omicron [141, 142]. So far, the antibody therapies reported to still be effective against all the current VOCs include VIR‐7831 (GlaxoSmithKline) and BRII‐196/BRII‐198 (Brii) [141, 142].

## Peptides targeting the CoV S2 subunit to inhibit membrane fusion

All members of the *Coronoaviridae* family share a similar mechanism of membrane fusion. The HR1 and HR2 elements in the S2 subunit are critical for forming the six‐helix bundle (6HB) and are highly conserved among different CoVs, indicating that they could serve as targets for developing broad‐spectrum antiviral drugs that inhibit membrane fusion. Peptides inhibiting 6HB formation and CoV infection have been designed and studied, such as CP1 derived from SARS‐CoV HR2, MERS‐HR2P derived from MERS‐CoV HR2, and 2019‐nCoV‐HR2P derived from SARS‐CoV‐2 HR2 [19, 143, 144]. EK1, a pan‐CoV fusion inhibitor derived from the HR2 of HcoV‐OC43 (Fig. 5C), exhibited broad‐spectrum antiviral activity against diverse CoVs including HCoV‐229E, HCoV‐OC43, HCoV‐NL63, SARS‐CoV, MERS‐CoV and SARS‐CoV‐2 [145]. The structural study showed that EK1 could block virus infection by snugly inserting into the hydrophobic groove formed by HR1 to form a 6HB architecture similar to that which mediates membrane fusion (Fig. 5C). Notably, EK1C4, a modified EK1 formed by conjugation of cholesterol at the C‐terminus, exhibited antiviral activity enhanced by more than 100‐fold against SARS‐CoV‐2 pseudovirus infection and S‐mediated membrane fusion [146]. EK1C4 also showed promising activity against infection with SARS‐CoV‐2, MERS‐CoV, HCoV‐NL63, HCoV‐229E and HCoV‐OC43 in mouse model [146]. Similarly, another HR2 sequence‐based lipopeptide fusion inhibitor, IPB02, has shown highly potent activity in inhibiting SARS‐CoV‐2 S protein‐mediated cell–cell fusion and pseudovirus infection [147].

## Conclusions and perspective

SARS‐CoV, MERS‐CoV and SARS‐CoV‐2 are representative newly emerging viruses that have had unprecedented global, social and economic impacts in the 21st century. Before the emergence of SARS‐CoV‐2, we had already gained significant structural insights into the infection, evolution and inhibition of highly pathogenic CoVs by studying SARS‐CoV and MERS‐CoV. These insights have played an important role in understanding the novel SARS‐CoV‐2 and successfully developing vaccines, antibodies, small‐molecule inhibitors and other antiviral agents in such a short period of time. Due to the focus on the S glycoprotein, this review did not include the many other important targets of current drug development efforts, such as viral non‐structural polymerases, viral proteases and host immune‐regulating molecules. The S glycoprotein has attracted immense attention as it is one of the most critical molecules for CoV infection, evolution and inhibition. For example, structural studies contributed to ideas for how to improve S glycoprotein stability for SARS‐CoV‐2 mRNA vaccine design [17]. Neutralizing antibodies that specifically target the S glycoprotein and block receptor binding and/or membrane fusion have also been approved for clinical application.

Since the outbreak of COVID‐19, multiple waves of increased infection and breakthrough infection of SARS‐CoV‐2 VOCs have occurred due to viral mutations arising that enhance receptor interaction and/or allow for escape from neutralizing antibodies. Additionally, it is likely inevitable that novel, highly pathogenic CoVs will continue to emerge. Thus, the broadly potent neutralizing agents and vaccines against SARS‐CoV‐2 VOCs and inhibitors with pan‐CoV antiviral activity are highly desirable. Compared to the NTD and RBD in the S1 subunit, the S2 subunit is more conserved in sequence and structure among different CoVs. Unfortunately, although S2‐directed antibodies usually exhibit broad binding, their neutralizing activity is lower than those targeting the NTD or RBD of the S1 subunit [148]. Notably, recent studies have shown that potent RBD‐directed antibodies are able to neutralize all SARS‐CoV‐2 VOCs, including Omicron, indicating that highly conserved and vulnerable sites within the S1 subunit do exist [149, 150]. It was also found that antibodies against SARS‐CoV can exhibit neutralizing activity against SARS‐CoV‐2. Further elucidation of these common epitopes using cross‐neutralizing antibodies by a combination of structural and functional studies would greatly facilitate immunogen design and vaccine development for inducing broadly neutralizing antibodies against present and future SARS‐CoV‐2 VOCs. Peptide inhibitors based on the HR1 or HR2 repeats in the S2 subunit have shown pan‐CoV antiviral activity. Recent studies also isolated S2‐targeting antibodies with neutralizing activities against SARS‐CoV, SARS‐CoV‐2 and MERS‐CoV [148]. These results collectively indicate that the S2 subunit remains an attractive target for developing potent pan‐CoV antibodies and vaccines.

## Conflict of interest

The authors declare no conflict of interest.

## Author contributions

SYQ, SYZ and JWG wrote the first draft of the manuscript and reviewed the literature; JWG and XQW reviewed and substantively edited the manuscript. All authors read and approved the final version.

## Data Availability

This review article does not present any novel data.
